# Benzoic and salicylic acids inhibit β-substituted alanine synthase 4;1 in common bean

**DOI:** 10.1093/plphys/kiaf485

**Published:** 2025-10-06

**Authors:** Zixuan Lu, Wojciech Witek, Milosz Ruszkowski, Barbara Imiolczyk, Nataliya Paulish, Jaya Joshi, Mariusz Jaskolski, Frédéric Marsolais

**Affiliations:** Agriculture and Agri-Food Canada, London Research and Development Centre, London, Ontario, Canada, N5V 4T3; Department of Biology, University of Western Ontario, London, Ontario, Canada, N6A 3K7; Department of Structural Biology of Eukaryotes, Institute of Bioorganic Chemistry, Polish Academy of Sciences, Poznan 61-704, Poland; Department of Structural Biology of Eukaryotes, Institute of Bioorganic Chemistry, Polish Academy of Sciences, Poznan 61-704, Poland; Department of Structural Biology of Eukaryotes, Institute of Bioorganic Chemistry, Polish Academy of Sciences, Poznan 61-704, Poland; Department of Structural Biology of Eukaryotes, Institute of Bioorganic Chemistry, Polish Academy of Sciences, Poznan 61-704, Poland; Agriculture and Agri-Food Canada, London Research and Development Centre, London, Ontario, Canada, N5V 4T3; Department of Biology, University of Western Ontario, London, Ontario, Canada, N6A 3K7; Department of Wood Science, University of British Columbia, Vancouver, British Columbia, Canada, V6T 1Z4; Department of Structural Biology of Eukaryotes, Institute of Bioorganic Chemistry, Polish Academy of Sciences, Poznan 61-704, Poland; Department of Crystallography, Faculty of Chemistry, Adam Mickiewicz University, Poznan 61-614, Poland; Agriculture and Agri-Food Canada, London Research and Development Centre, London, Ontario, Canada, N5V 4T3; Department of Biology, University of Western Ontario, London, Ontario, Canada, N6A 3K7

## Abstract

The nutritionally essential sulfur amino acids, methionine and cysteine, are present at suboptimal levels in legumes, such as common bean (*Phaseolus vulgaris* L.). β-Substituted alanine synthase 4;1 (BSAS4;1) is the major isoform of cytosolic cysteine synthase present in the developing seeds of common bean. There is evidence that in addition to cysteine, this enzyme is also involved in the biosynthesis of the non-proteinogenic amino acid *S*-methylcysteine, which accumulates in the form of a γ-glutamyl dipeptide. Here, we report the high-resolution structure of recombinant BSAS4;1. Unexpectedly, the crystal structure showed the presence of a molecule of benzoic acid near the active site, which appeared to have been co-purified from *Escherichia coli*. Kinetic analysis indicated that benzoic acid acts as a competitive inhibitor of BSAS4;1 with respect to *O*-acetylserine. IC_50_ values for benzoic acid and the structurally related salicylic acid were both equal to 0.6 mm. Using developing cotyledons grown in vitro, quantification of the incorporation of ^13^C_3_- and ^15^N-labeled serine into cysteine and downstream metabolites indicated that benzoic acid effectively inhibited cysteine biosynthesis in vivo at a concentration of 1.2 mm. The results of experiments tracking the incorporation of ^13^C-labeled sodium thiomethoxide provided further evidence that BSAS4;1 may be involved in the formation of free *S*-methylcysteine, through the condensation of *O*-acetylserine with methanethiol.

## Teaser

Benzoic and salicylic acids inhibit β-substituted alanine synthase 4;1 (BSAS4;1) in *Phaseolus vulgaris* L. to affect the metabolism of sulfur-containing amino acids and their metabolites.

## Introduction

**Figure 1.**
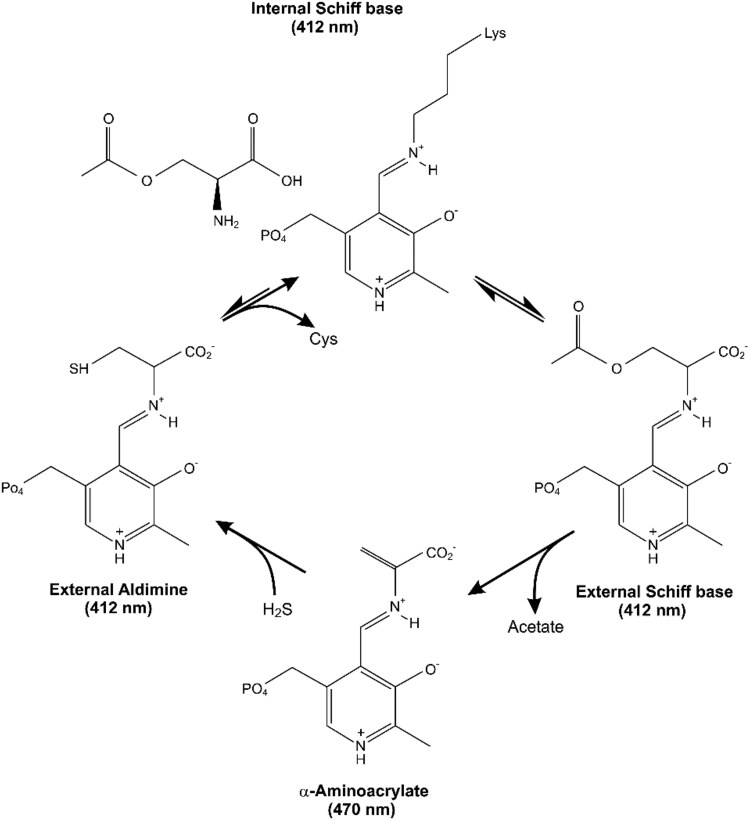
Mechanism of cysteine synthase.

In microorganisms and plants, the enzyme cysteine synthase (EC 2.5.1.47) is responsible for the assimilation of inorganic sulfur into an organic compound (Kopriva et al. 2024). Cysteine synthase is a member of the pyridoxal 5′-phosphate (PLP)-dependent β-substituted alanine synthase (BSAS) family (Yi and Jez 2012). The enzyme catalyzes a β-replacement, exchanging the acetyl moiety of the activated form of serine, *O*-acetyl-L-serine (OAS), for sulfide, producing L-cysteine (Bonner et al. 2005). In the resting state of the enzyme, the PLP prosthetic group is bound to the ε-amino group of the side chain of a conserved lysine residue, forming an internal Schiff base (Fig. 1). Upon substrate binding, the α-amino group of OAS reacts by displacing the lysine side chain to form an external Schiff base (Fig. 1). This allows the conserved lysine residue to act as a general base in the elimination of the acetate moiety from OAS, which leads to an α-aminoacrylate intermediate (Fig. 1). Next, a sulfide nucleophile reacts with the latter moiety to form an external aldimine with a thiol group (Fig. 1). To close the catalytic cycle, the conserved lysine reacts with the aldimine intermediate to release the cysteine product and regenerate the internal Schiff base.

The BSAS proteins belong to the family of PLP-dependent fold type II enzymes (Schneider et al. 2000), which exist as homodimers (Bonner et al. 2005). In plants, different genes encode cysteine synthase isoforms in plastids, mitochondria, and cytosol (Hell and Wirtz 2011). Cysteine synthase has the capacity to form a cysteine synthase complex, or cysteine regulatory complex, with the enzyme serine acetyltransferase (Wirtz and Hell 2006), which is important for the regulation of cysteine synthase. A major form of the plant complex contains 2 serine acetyltransferase trimers and 2 cysteine synthase dimers (Wirtz et al. 2010); however, other assemblies have also been observed (Kumaran et al. 2009). Serine acetyltransferase trimers interact head to head through their N-termini, whereas serine acetyltransferase interacts with cysteine synthase through its C-terminal region, so that different associations are possible (Kaushik et al. 2017). Complex formation activates serine acetyltransferase and inhibits cysteine biosynthesis (Yi et al. 2013). Sulfide stabilizes the complex. Cysteine synthase is present in excess of serine acetyltransferase (Hesse and Hoefgen 2008). Accumulation of *O*-acetylserine during sulfur deficiency dissociates the complex. The carboxyl end of serine acetyltransferase binds to the active site of cysteine synthase with high affinity (Kumaran et al. 2008). Under these conditions, substrate access is achieved through a competitive allosteric mechanism (Kaushik et al. 2021). As this mechanism is unique to microorganisms and plants, cysteine biosynthesis has recently emerged as a target for novel antibiotics and herbicide development (Schnell et al. 2015; Hicks and Mullholland 2018; Foletto-Felipe et al. 2023; Ben-Shushan et al. 2024). However, in the last decade, cysteine synthases from protozoans such as *Leishmania* sp. and *Trypanosoma* sp., have also been used as potential drug targets (Fyfe et al. 2012; Sowerby et al. 2023). This is especially interesting given the fact that cysteine synthases, despite usually low sequence identities, share a similar, conserved fold. Thus, information about inhibitors of cysteine synthases from one group of organisms can be applied to enzymes from other species (Hicks and Mullholland 2018). Another upside of targeting cysteine biosynthesis is the absence of de novo cysteine biosynthesis in mammals, which synthesize cysteine from methionine. This enhances the selectivity of any drugs targeting cysteine biosynthesis by minimizing the risk of nonspecific interactions with human enzymes.

The concentration of the nutritionally essential sulfur amino acids methionine and cysteine is sub-optimal in the seeds of common bean (*Phaseolus vulgaris*), as in other grain legumes (De Ron et al. 2015). However, a unique characteristic of several *Phaseolus* and *Vigna* species is the accumulation in their seeds of the nonprotein amino acid *S*-methylcysteine (Baldi and Salamini 1973), principally as a γ-glutamyl dipeptide (Saboori-Robat et al. 2019). Results of stable isotope incorporation studies have shown the existence of 2 distinct *S*-methylcysteine biosynthetic pathways, involving either OAS or homoglutathione as precursor (Joshi et al. 2019). BSAS4;1, a cytosolic cysteine synthase, was identified as the likely enzyme involved in the formation of free *S*-methylcysteine through the condensation of OAS and methanethiol. The corresponding transcript is abundantly expressed in developing seeds of *P. vulgaris*.

In the present study, we sought to further characterize BSAS4;1. The recombinant *P. vulgaris* enzyme (PvBSAS4;1) was crystallized, and its high-resolution structure was solved using synchrotron-radiation X-ray diffraction data. The crystal structure showed the unexpected presence of a molecule of benzoic acid near the active site. Following this observation, the effect of benzoic acid as an inhibitor was characterized, both in vitro and in vivo. Additional evidence from incorporation of a stably labeled precursor further supports the hypothesis that OAS and methanethiol may be combined to form *S*-methylcysteine.

## Results

### Overview of the β-substituted alanine synthase 4;1 structure

PvBSAS4;1 is a PLP-dependent enzyme (EC 2.5.1.47) belonging to the cytosolic clade II cysteine synthases (Yi and Jez 2012; Joshi et al. 2019). Members of this subgroup normally utilize OAS and sulfide as substrates in the process of de novo cysteine biosynthesis and, therefore, are named *O*-acetylsulfhydrylases (OASSs) or cysteine synthases (CysM and CysK) (Tao et al. 2024). PvBSAS4;1 has high catalytic activity with OAS and sulfide as substrates, comparable with the activity of cysteine synthases from other crops (Joshi et al. 2019).

**Figure 2.**
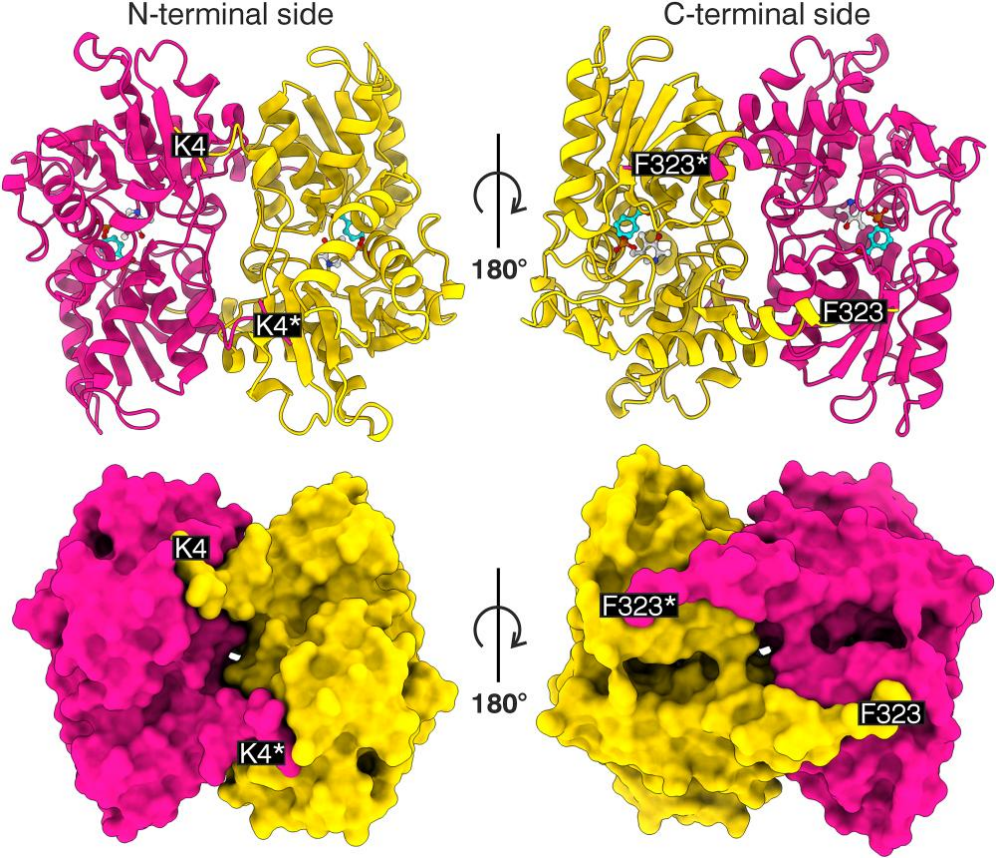
Overview of the PvBSAS4;1 homodimer structure. The top section shows ribbon diagrams and the bottom shows surfaces of the 2 sides of the enzyme, viewed from the direction of the N-terminal (K4) and C-terminal (F323) residues. Asterisks denote the second subunit of the dimer.

The crystal structure of PvBSAS4;1 was solved in the *P*4_1_2_1_2 space group, with one protein chain in the asymmetric unit (Table 1). The full-length amino acid sequence of the enzyme comprises 324 residues (UniProt ID: V7BIJ9). However, the experimental electron density covers the range between Lys4 and Phe323. The biological assembly of PvBSAS4;1 is a homodimer, in which the 2 subunits are related by a crystallographic 2-fold axis, and cling to each other using their N- and C-termini. The location of the termini allows for the distinction between 2 sides of the homodimer, namely the N- and C-terminal sides (Fig. 2, Supplementary Fig. S1). Analyses of the homodimer surface potential distribution revealed that the C-terminal side contains 2 pockets of high positive charge, which form the entrances to the 2 active sites (Fig. 2).

**Table 1.** Crystallographic parameters and data collection and refinement statistics for PvBSAS4;1

Data collection	
Beamline	EMBL P13/Petra III, DESY Hamburg
Wavelength (Å)	1.0332
Temperature (K)	100
Space group	*P*4_1_2_1_2
Unit cell parameters (Å) *a* *=* *b*, *c*	69.19, 186.43
Oscillation range (°)	0.2
Number of images	1,000
Resolution range (Å)	47.30 to 1.60 (1.70 to 1.60)
Reflections collected/unique	860,835/60,777
Completeness (%)	99.9 (99.5)
Multiplicity	14.16 (6.31)
Wilson *B* (Å^2^)	18.9
*R*_merge_ (%)	6.8 (156.6)
*R*_meas_ (%)	7.0 (162.6)
<*I*/*σ*>	20.97 (1.65)
CC_1/2_ (%)	100.0 (73.6)
Refinement	
Unique/test reflections	59,561/1,216
*R*_work_/*R*_free_ (%)	14.1/16.8
Protein chains in ASU	1
Matthews coefficient (Å^3^/Da)/solvent (%)	3.23/61.95
ADP model	TLS + individual isotropic
<*B*> for protein/ligands/water (Å^2^)	33.0/38.6/50.7
Protein/ligand/solvent/atoms	2,446/38/271
Rmsd bonds (Å)/angles (°)	0.009/0.92
Ramachandran plot (%) favored/allowed/outliers	97/3/0
PDB code	9RJ1

Values in parentheses correspond to the last resolution shell.

Per analogy to the similar structure of *Arabidopsis thaliana* OASS (AtOASS, UniProt ID: P47998, PDB ID: 1Z7W), which shares 69% sequence identity with PvBSAS4;1, 2 functional α/β domains can be distinguished, using the previously established terminology (Bonner et al. 2005). The smaller, N-terminal or substrate-binding domain covers the range between Val47 and Glu152, and is folded as 4 β-strands flanked by 4 α-helices. The larger, C-terminal or PLP-binding domain spans 2 residue ranges, namely Lys4-Ser46 and Asn153-Phe323. Similar domains are present in other members of type II PLP-dependent OASSs, even those that are not closely related to PvBSAS4;1, indicating the importance of this architecture for cofactor binding and catalysis. Details about sequence and structural similarities between PvBSAS4;1 and its homologs are available in Supplementary Fig. S2 and Supplementary Table S1.

### Unexpected electron density in the active site

**Figure 3.**
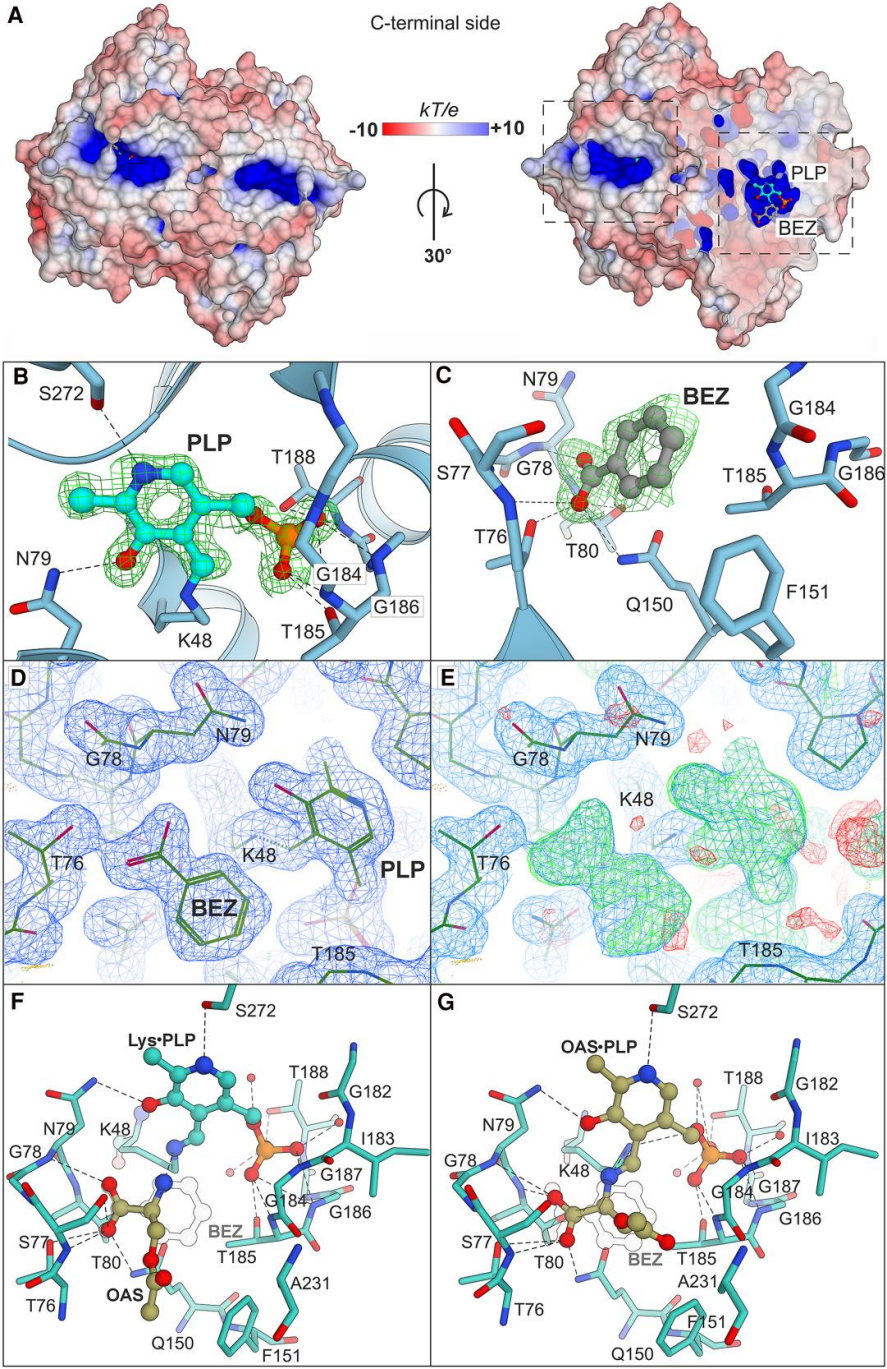
Active sites and the main ligands in the PvBSAS4;1 structure, seen from the C-terminal side. Panel **(A)** shows the location of the ligand-containing active sites in the context of the surface potential colored as in the scale bar, highlighted by dashed rectangles. The cross-section through the enzyme's surface is 60% transparent. Panels **(B)** and **(C)** illustrate the molecular interactions between the protein residues and the PLP and BEZ ligands, respectively. Residues within a 4-Å range from PLP and a 6-Å range from BEZ are shown. The polder map (green mesh) for PLP is contoured at 3.0*σ*, while the map for BEZ is contoured at 2.7*σ*. **(D)** Unbiased composite OMIT map contoured at 1.0*σ*, calculated without the presence of any ligands. **E)** 2Fo-Fc (blue, 1.0*σ*) and Fo-Fc (green, 3.0*σ*) maps after 6 cycles of initial refinement. **F** and **G)** Results of in silico docking of free OAS to PvBSAS4;1 in the internal aldimine state **(F)** and PLP–OAS external aldimine to the enzyme with free Lys48 **(G)**. The BEZ molecule from the crystal structure is outlined. Note how the carboxyl group of OAS aligns with that of BEZ.

The overall fold of PvBSAS4;1 resembles that of other OASSs, which belong to the type II fold, one of the 5 folds of PLP-binding enzymes described in total (Eliot and Kirsch 2004). The active sites of the fold type II enzymes are entirely constituted of residues from one protomer, hosting a PLP molecule bound to a conserved lysine residue (Fig. 3). The positively charged active site of the present PvBSAS4;1 structure contains the PLP prosthetic group and a molecule of benzoic acid (Fig. 3A). Both ligands interact with the active site residues, which are conserved among bacteria, plants, fungi, and protozoa (Supplementary Fig. S2). PLP is stabilized by a covalent bond to Lys48, creating an internal aldimine. Other interactions occur via hydrogen bonding between the PLP's phosphate and the backbone amides of Gly184 (2.8 Å), Thr185 (2.8, 3.4 Å), Gly186 (2.8 Å), and Thr188 (2.9 Å). The phosphate moiety is also hydrogen-bonded to the side chains of Thr185 (2.7 Å) and Thr188 (2.6 Å). The pyridine ring interacts with the side chains of Asn79 (2.9 Å) and Ser272 (2.9 Å) via the O3 and N1 atoms, respectively (Fig. 3B).

The benzoate (BEZ) molecule is located ∼3.7 Å away from the PLP ring, with its carboxylic end facing the protein core and the phenyl ring pointing toward the entrance to the active site (Fig. 3C). Its carboxylate interacts with the backbone amides of Ser77 (3.0 Å), Asn79 (3.1 Å), and Thr80 (2.9 Å), as well as with the side chains of Thr76 (2.5 Å), Thr80 (3.0 Å), and Gln150 (3.0 Å). The phenyl ring is stabilized by hydrophobic interactions with the backbone of Gly184 (3.8 Å), and side chains of Phe151 (3.7 Å) and Thr185 (3.9 Å), which create a ligand-binding cavity (Fig. 3C). Unbiased maps, i.e. a composite omit map and a difference Fo-Fc map showing electron density prior to modeling of the BEZ molecule are shown in Fig. 3, D and E.

The structure of PvBSAS4;1 includes a few more accidental ligands, attracted from the crystallization buffer, such as 2 molecules of glycerol, a sodium cation, and a chloride anion, all bound on the enzyme surface.

We were unable to obtain an experimental structure of the PvBSAS4;1-OAS complex via co-crystallization or soaking. This difficulty is likely attributable to the reversible and dynamic nature of the initial half-reaction, i.e. the formation of PLP–OAS external aldimine. Therefore, we employed in silico docking to identify residues involved in substrate interaction. In the best-scoring pose, free OAS bound to PvBSAS4;1 as internal aldimine at Lys48, interacts through its carboxylate group (Fig. 3F) analogously to BEZ (Fig. 3C). The amino group projects toward the PLP reactive center, consistent with the proposed reaction mechanism (Fig. 1). The acetyl group is positioned near Tyr75, Ile127, Ile130, and Phe151; however, its oxygen atoms do not participate in hydrogen bonding. In the PLP–OAS external aldimine form, the overall orientation of the OAS moiety remains largely unchanged, with the exception of the acetyl group, which points toward Ala231 (Fig. 3G).

Molecular dynamics (MD) simulations (in 2 replicas) were run for both aforementioned complexes modeled as dimers. Free OAS docked to PvBSAS4;1 in the PLP-Lys internal aldimine form dissociated between 100 and 610 ns timepoints, as reflected by the number of protein–ligand contacts (Supplementary Fig. S3A). In contrast, the PLP–OAS external aldimine remained bound in the active site throughout the entire 1 µs simulation in all cases (Supplementary Fig. S3B). Consistently, the PLP–OAS external aldimine formed approximately 20 contacts (hydrogen bonds, hydrophobic interactions, ionic interactions, and water bridges) with the protein at any given time, nearly thrice as many as free OAS. The interactions involving the carboxylate group of PLP–OAS were maintained for roughly half of the simulation time, whereas those formed by the PLP moiety persisted for nearly the entire duration (Supplementary Fig. S4).

### Benzoic and salicylic acids inhibit the activity of PvBSAS4;1 in vitro

**Figure 4.**
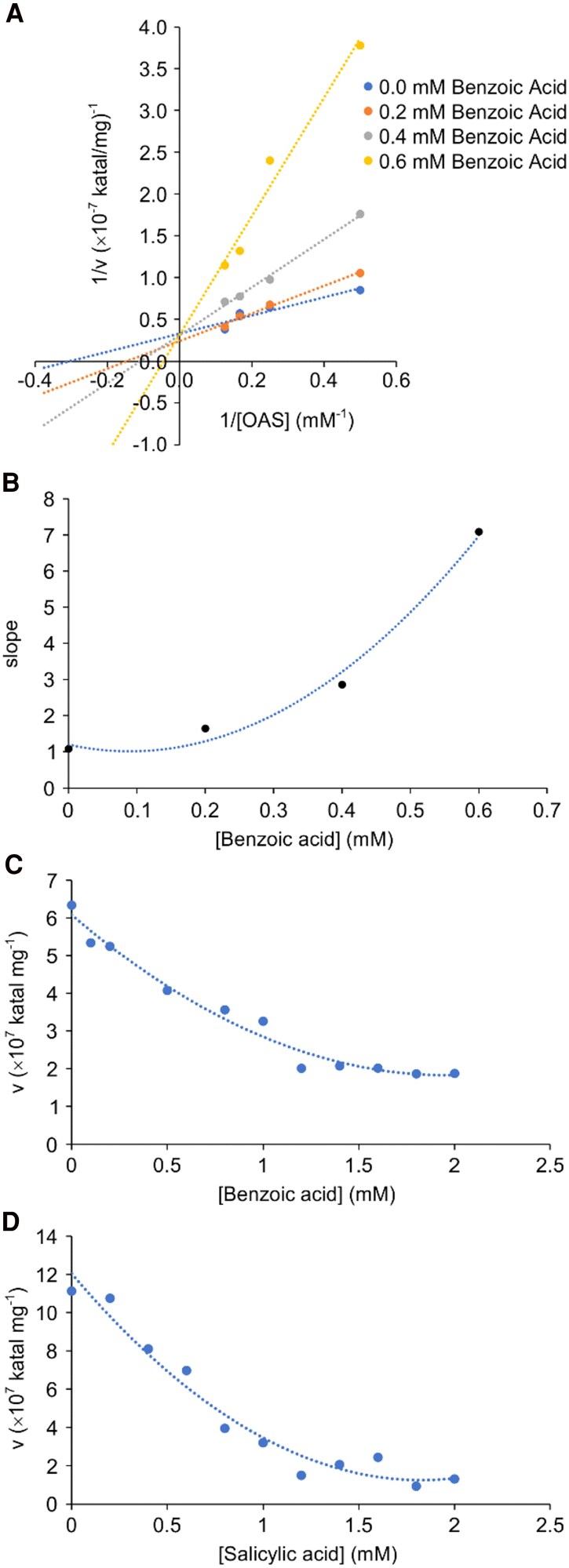
Characterization of the inhibition of PvBSAS4;1. **A)** Lineweaver–Burk plot of the reciprocal of the rate of the reaction vs. the reciprocal of the concentration of *O*-acetylserine at different benzoic acid concentrations. **B)** Replot of the slope vs. benzoic acid concentration. Plots of the rate of the reaction vs. concentration of benzoic acid **(C)** and salicylic acid **(D)** in enzymatic assays with *O*-acetylserine and sodium sulfide as substrates, average of *n* = 2. See text for explanations.

To characterize the inhibition of BSAS4;1 by benzoic acid, the recombinant enzyme was assayed at different concentrations of OAS and inhibitor, and a fixed concentration of sodium sulfide. A Lineweaver–Burk plot was used to determine the type of inhibition of PvBSAS4;1 by benzoic acid, with the reciprocal of the reaction rate on the *y* axis and the reciprocal of OAS concentration on the *x* axis, at different inhibitor concentrations. The lines intersect at the ordinate, meaning that benzoic acid acts as a competitive inhibitor with respect to OAS (Fig. 4A). Addition of benzoic acid did not change the rate of the reaction but affected the apparent *K*_m_ (or *K*_s_  _app_), a symptom that is diagnostic of competitive inhibition. The replot of the slope versus inhibitor concentration is parabolic (Fig. 4B), indicating multisite inhibition interactions (Segel 1975; Leskovac 2003). As expected, the replot of *K*_s_  _app_ versus the concentration of inhibitor and the Dixon plot of the reciprocal of the rate versus the inhibitor concentration were also parabolic (Supplementary Fig. S5, A and B). It is not possible to determine *K*_i_ from the Dixon plot, because it is not linear. Instead, *K*_i_  _slope_ is calculated from the slope of the reciprocal plot, and is a complex function of *K*_i_ that varies with [I]. *K*_i_  _slope_ was calculated according to the following equation (Segel 1975):


$$\mathrm{Slope}\;1/s=K_{s}/lV_{\max}(1+[I]/K_{i\;\mathrm{slope}})$$

The replot of the reciprocal of *K*_i_ slope versus inhibitor concentration was parabolic, suggesting more than 2, possibly 4 independent binding sites (Supplementary Fig. S5C).

The inhibitory effect of benzoic acid and other molecules was compared. A range of concentrations between 10 μM and 2 mm of benzoic acid was used in enzymatic assays with OAS and sodium sulfide as substrates (Fig. 4C). The IC_50_ value of benzoic acid was equal to approximately 0.6 mm. Salicylic acid, tyrosine, and phenylalanine have similar chemical structures to benzoic acid and were tested as inhibitors. Salicylic acid acted as an inhibitor of PvBSAS4;1 while tyrosine and phenylalanine did not. Figure 4D shows the result of the enzymatic assays with salicylic acid. The IC_50_ of salicylic acid was also equal to 0.6 mm.

### Inhibition of PvBSAS4;1 by benzoic acid in vivo

**Figure 5.**
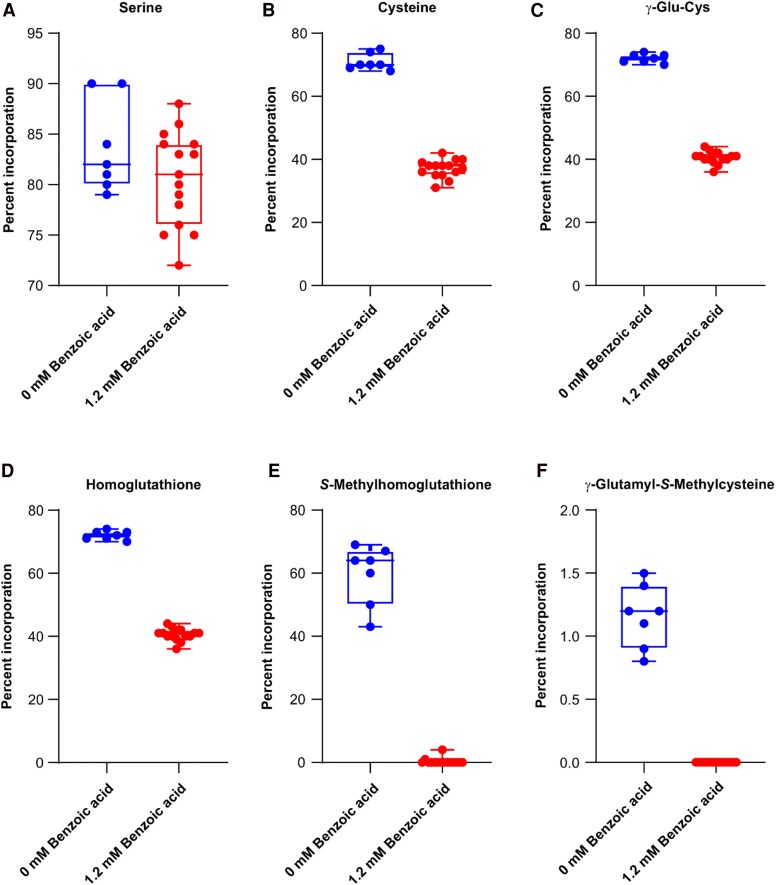
Box plots showing uptake and incorporation of ^13^C_3_-, ^15^N-labeled serine into downstream metabolites in developing cotyledons grown in vitro in the presence or absence of 1.2 mm benzoic acid. **A)** Serine. **B)** Cysteine. **C)** γ-Glutamyl-cysteine (γ-Glu-Cys). **D)** Homoglutathione. **E)**  *S*-Methylhomoglutathione. **F)** γ-Glutamyl-*S*-methylcysteine. Center line of the boxplot represents the median. The first and third quartiles represent the boundaries of the box. All data points are displayed. The boundary of the lower whisker is the minimum value of the dataset. The boundary of the upper whisker is the maximum value of the dataset.

To determine if benzoic acid acts as an inhibitor in vivo, developing cotyledons were grown in its presence and fed with ^13^C_3_- and ^15^N-labeled serine. Based on the appearance of cotyledons after 48 h of incubation with a range of benzoic acid concentrations between 0 and 1.2 mm at 0.2 mm intervals, all cotyledons were healthy and had a consistent weight increase across this range. Thus, 1.2 mm benzoic acid was chosen for in vivo assay to better observe the inhibitory effect. The percentage of isotope incorporation was measured by high-resolution LC–MS-MS. Samples of 6 cotyledons were analyzed by LC–MS. Based on the result of the MS analysis, uptake of labeled serine was comparable in the control and inhibitor-treated samples (Fig. 5A). The percentage incorporation in cysteine was reduced by approximately half (Fig. 5B), indicating that benzoic acid is active as an inhibitor of PvBSAS4;1 in vivo. The percentage of incorporation in the downstream metabolites, γ-glutamylcysteine and homoglutathione, was reduced by approximately the same factor (Fig. 5, C and D). Interestingly, incorporation into 2 metabolites that incorporate the *S*-methylated form of cysteine, *S*-methylhomoglutathione and γ-glutamyl-*S*-methylcysteine, was completely abolished in the presence of benzoic acid (Fig. 5, E and F). In this experiment, *S*-methylcysteine was not quantified, as free *S*-methylcysteine peaks overlapped with those arising from the dissociation of γ-glutamyl-*S*-methylcysteine.

### In vivo evidence for incorporation of methanethiol into *S*-methylcysteine

**Figure 6.**
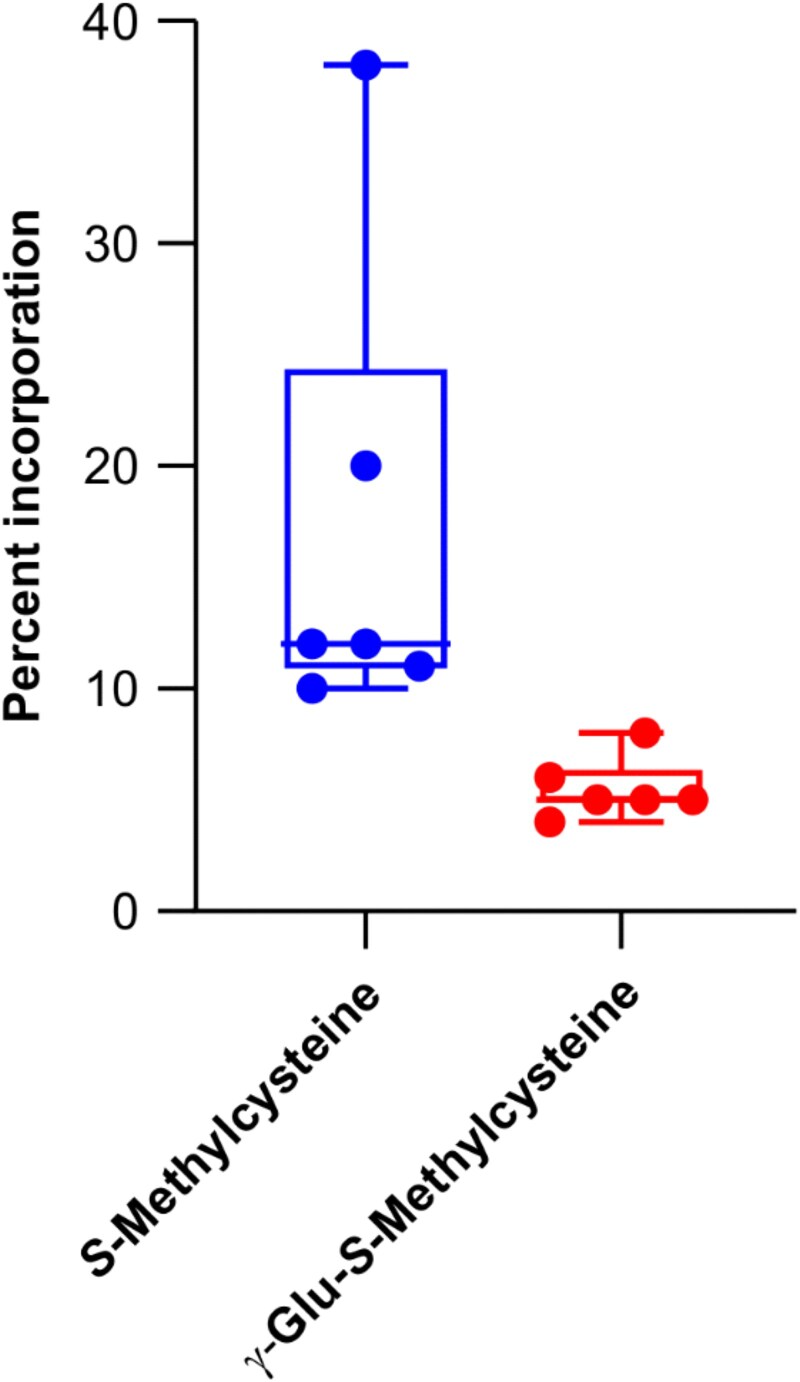
Box plot showing incorporation of ^13^C-labeled methanethiol into *S*-methylcysteine and γ-glutamyl-*S*-methylcysteine in developing cotyledons grown in vitro. Center line of the boxplot represents the median. The first and third quartiles represent the boundaries of the box. All data points are displayed. The boundary of the lower whisker is the minimum value of the dataset. The boundary of the upper whisker is the maximum value of the dataset.

To provide additional information about the possible role of PvBSAS4;1 in forming *S*-methylcysteine in vivo, an experiment was conducted with ^13^C-labeled sodium thiomethoxide as precursor, and incorporation into target compounds was analyzed by LC–MS. In this experiment, the chromatography conditions were modified to accurately separate *S*-methylcysteine from γ-glutamyl-*S*-methylcysteine, by increasing the time in solvent A (0.1% formic acid) from 0.5 to 1.25 min and substituting the Zorbax RRHD Eclipse Plus C18 column with a Zorbax SB-Aq RRHD Threaded Column (Agilent Technologies, Mississauga, Ontario, Canada). The results are presented in Fig. 6. Incorporation of isotope-labeled substrate was measured in *S*-methylcysteine, followed by γ-glutamyl-*S*-methylcysteine. No significant incorporation was detected in *S*-methylhomoglutathione, γ-glutamylcysteine, or homoglutathione.

## Discussion

Structural topology of the PvBSAS4;1 protein resembles that of other PLP-utilizing OASSs, including plant, bacterial, and amoebozoal homologs (Bonner et al. 2005; Schnell et al. 2007; Chinthalapudi et al. 2008; Yi et al. 2012; Smith et al. 2022). The conserved fold reflects its importance for supporting the proper enzyme function, primed by PLP bound in the 2 active sites of the homodimer. The presence of 2 active sites in this homodimer may explain at least in part the parabolic shape of the Dixon plot and its slope replot (Fig. 4B), possibly as a result of the activation and cooperation of the active sites, induced by the structural changes of the enzyme taking place in the course of the catalytic reaction. However, to test this speculation, additional structural studies are necessary to capture the potential conformational changes induced by ligand binding.

The rationale behind the present structural study is related to the work published by Joshi et al. (2019), who studied the biosynthesis of sulfur-containing compounds important for nutritional improvement of common bean, using isotopic tracking and mass spectrometry. One of those important compounds is the nonproteinogenic amino acid *S*-methylcysteine, which is synthesized in developing seeds from OAS and methanethiol. Therefore, we decided to co-crystallize PvBSAS4;1 with 5 mm OAS and 20 mm sodium thiomethoxide, acting as an analog of methanethiol, to find its binding site. Unfortunately, those crystallization trials, as well as equivalent soaking experiments, were unsuccessful.

**Figure 7.**
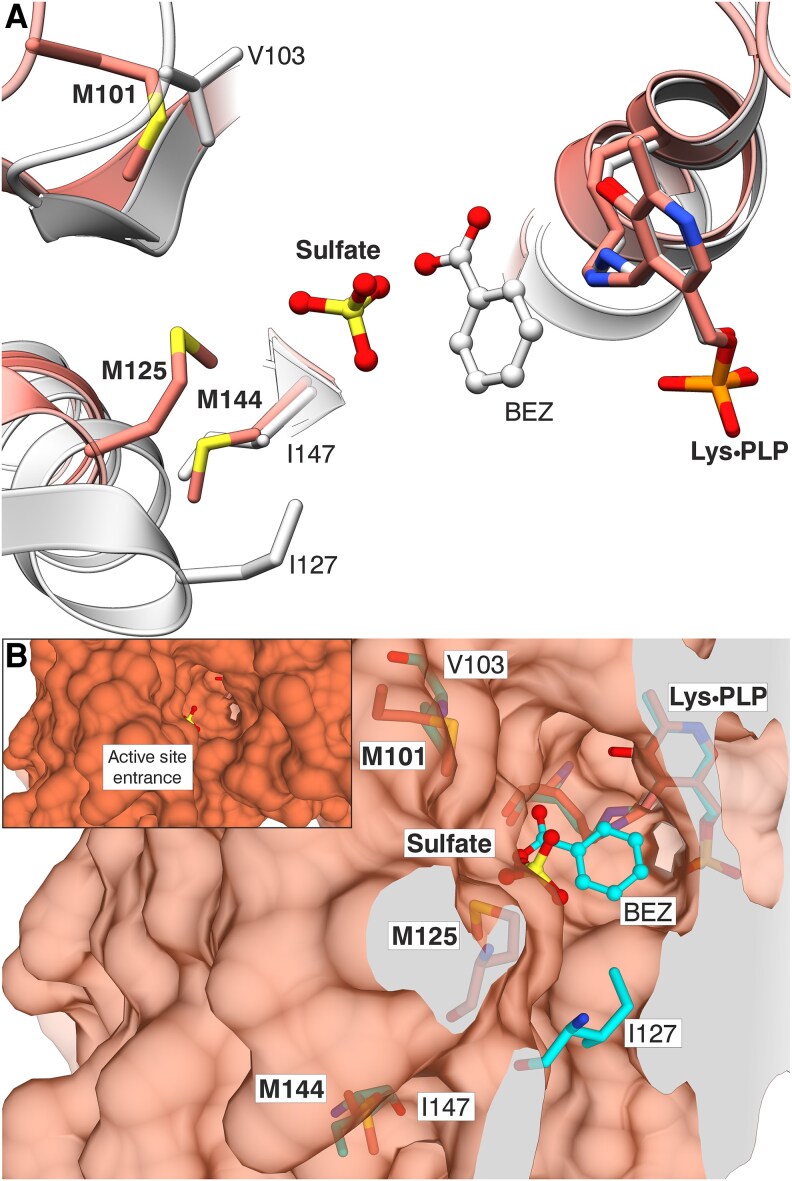
Structural comparison of the active site entrance in AtOASS. AtOASS is shown in coral (PDB ID: 1Z7W) while PvBSAS4;1 in cyan. Bolded labels indicate residues (and sulfate) from the AtOASS structure, while the remaining ones are from PvBSAS4;1. To enhance the clarity in Panel **(A)**, secondary structure elements from the front and back planes have been removed. Panel **(B)** shows the environment around the active site in AtOASS created by the methionine residues, which are substituted by Val and Ile residues in PvBSAS4;1. AtOASS surface is 50% transparent. The inset in **(B)** shows a zoom-out view with nontransparent surface.

To investigate the potential evolutionary adaptation of PvBSAS4;1 for methanethiol processing, we superposed its structural model onto that of AtOASS (PDB ID: 1Z7W), obtaining a root-mean-square deviation of 0.79 Å for 289 superposed Cα atoms. Interestingly, PvBSAS4;1 retains only a single methionine residue (Met99), corresponding to Met97 in AtOASS. The remaining methionine residues at positions 101, 125, and 144 in AtOASS are replaced in PvBSAS4;1 by Val103, Ile127, and Ile147, respectively (Fig. 7). These methionines in AtOASS form distinct clefts that lead to the enzyme's active site. Given that methanethiol is chemically part of the sulfur-containing side chain of methionine, the absence of these bulky methionine residues in PvBSAS4;1 may represent an adaptive loss. These substitutions potentially create small hydrophobic niches along the substrate channel, which could transiently bind and relay free methanethiol on its passage to the active site. Such structural changes may facilitate substrate sequestration and delivery, offering a plausible evolutionary advantage for efficient methanethiol utilization. In addition, the 3 hydrophobic residues in PvBSAS4;1 could likely contribute to the stabilization of the methyl group of methanethiol.

Although we did not obtain the desired substrate complex of PvBSAS4;1, we found a distinct patch of electron density corresponding to benzoic acid, bound in the active site in a position usually occupied by the OAS substrate. It was an unexpected observation, because benzoic acid, a well-known food preservative inhibiting the growth and survival of microorganisms, had never been added at any step of the protein purification or crystallization processes. The initial hypothesis explaining its presence in the active site was that it might have been added to the crystallization screen as a preservative. However, the manufacturer, whom we queried about this puzzle, claimed that BEZ was not used in the production process of their screen solution. Therefore, we assume that benzoic acid must have been co-purified from *Escherichia coli* culture. The sequestration and retention of BEZ in the enzyme's active site suggest a high binding affinity.

As we were not able to solve the structure of a PvBSAS4;1/OAS complex, we first attempted to build such a model using other complex structures (PDB ligand ID: OAS) available in the PDB (4ORE, 4ZU1, 4ZU6, 5DBE, 8B9Y). It was a puzzling observation that when the complexes were Cα-superposed, the positioning of OAS was different in those 5 homologous structures (Supplementary Fig. S6, Supplementary Table S2). Intrigued by that, we analyzed the original experimental electron density maps as well as the interactions that the OAS ligands were proposed to make with the proteins. Our analysis revealed that the electron density maps of the deposited structures do not fully support the proposed models and that there are doubts about the biochemical correctness of protein–ligand interactions. These include: (i) inverted orientation of OAS in the active site; (ii) modeling OAS on the protein surface despite no evidence from the electron density; and (iii) absence of the key residues as interaction partners for OAS in the active site (Supplementary Fig. S7). We would like to stress that crystallographic modeling, especially of protein–ligand complexes, must always be supported by experimental electron density and conform to the rules of stereochemistry (Dauter et al. 2014). As a final conclusion, the existing experimental evidence is insufficient to support the modeling of the catalytically relevant OAS ligand in the PDB structures 4ORE, 4ZU1, 4ZU6, 5DBE, and 8B9Y.

With this conclusion in mind, we docked free OAS to PvBSAS4;1 in the internal aldimine form, and the PLP–OAS external aldimine to PvBSAS4;1 with Lys48 in free amine form. In both cases, the ligand carboxylates occupied similar positions to those of the bound BEZ molecule. More importantly, we found the amine of free OAS reaching toward the C4′ atom of PLP, therefore presenting a chemically valid positioning for the first half-reaction. The MD simulations suggested that the complex with free OAS is not stable, in contrast to the complex with PLP–OAS. In the latter case, only the acetyl group displayed a dynamic behavior, while the rest of the molecule remained stably bound to the protein. Although the MD simulations highlighted the role of PLP in anchoring OAS, the details of the β-replacement of the acetyl group for thiomethyl moiety remain unresolved.

Since BEZ is widely produced in plants, we decided to test its inhibitory potential on PvBSAS4;1 activity. The co-purification hypothesis aligns with the IC_50_ value of BEZ for PvBSAS4;1, which is 0.6 mm, and with the competitive nature of the inhibitor with respect to OAS. Salicylic acid, also widely distributed and involved as a secondary messenger in plant defense (Huang et al. 2020), inhibited the enzyme with a similar IC_50_ value. The presence of BEZ in the active site of PvBSAS4;1 raised the question of what types of compounds could bind in this area. A comparison of our structural results with other available OASS complexes reveals that BEZ occupies a conserved region, corresponding to ^76^TSGNT^80^ in PvBSAS4;1 (Fig. 3C). This region supports binding of a variety of chemical molecules, such as sulfate ions (PDB: 1Z7W), methionine (1Z7Y, 4JBL), cysteine (3VC3, 3BM5), isoleucine (4IL5), 2-methyl-2,4-pentanediol (MPD; 2Q3B), or citric acid (CIT; 2V03, 7N2T). The role of this conserved pentapeptide was investigated by mutagenesis of the ^74^TSGNT^78^ sequence in AtOASS (Bonner et al. 2005). The kinetic studies of AtOASS indicated that Thr74 and Ser75, which correspond to Thr76 and Ser77 in PvBSAS4;1, participate in the incorporation of sulfur atoms into cysteine by binding its carboxyl group and are, therefore, crucial for catalysis. Further analyses demonstrated that the 4 T74A/S and S75A/N mutants displayed over 10-fold increase of the *K*_M_ value for sodium sulfide, and both the T74A and T74S mutants displayed slower reaction rates with OAS and sulfide, compared to the wild type. The mutants also have reduced the *k*_cat_ turnover values, and their overall catalytic efficiency for sodium sulfide is decreased up to 1,000-fold (Bonner et al. 2005).

The results of incorporation experiments confirmed that BEZ acts as an inhibitor of cysteine biosynthesis in vivo at a similar concentration as in vitro. Reductions in incorporation of stably labeled serine into cysteine, and the downstream metabolites γ-glutamylcysteine and homoglutathione, were comparable. Interestingly, incorporation into 2 other metabolites, γ-glutamyl-*S*-methylcysteine and *S*-methylhomoglutathione, was completely abolished. Past results highlighted a distinct branch of the *S*-methylcysteine biosynthetic pathway involving *S*-methylhomoglutathione as an intermediate (Joshi et al. 2019). The present results suggest that BEZ further inhibits an additional step within this pathway. Other PLP-dependent enzymes of plant sulfur amino acid biosynthesis are inhibited by phenolic compounds, such as cystathione γ-synthase by molecules having a phenyl benzamide moiety (Bloch et al. 2021). The results obtained with ^13^C-labeled sodium thiomethoxide further support the notion that BSAS4;1 is involved in the formation of free *S*-methylcysteine through condensation of OAS and methanethiol.

The ability of PvBSAS4;1 to bind small-molecule ligands opens the possibility for rational design of molecules that might act as cysteine-metabolism modulators in *P. vulgaris*, particularly as competitive inhibitors. That approach was proved successful by Poyraz et al. (2013), who synthesized potent thiazolidine inhibitors (IC_50_ = 19 nm) of a homologous *Mycobacterium tuberculosis* CysK1 enzyme (PDB: 3ZEI). The thiazolidine inhibitor interacts with the ^71^TSGNT^75^ region of CysK1 via its corresponding phenyl moiety. This is just one example of recent advances in cysteine biosynthesis inhibition, which in most studies targeted bacterial homologs of PvBSAS4;1. Other examples comprise pentapeptide inhibitors, cyclopropane carboxylic acid derivatives, urea derivatives, and many others, including 1,2,3-triazoles (Tao et al. 2024), which are also known as inhibitors of histidine biosynthesis.

## Materials and methods

### Recombinant protein purification

Protein purification was conducted separately in 2 different laboratories; therefore, a general method is described below, and protocol differences are summarized in Table 2.

**Table 2.** Summary of conditions and reagents used for BSAS4;1 purification

	Enzymatic experiments	Structural studies
*E. coli* strain	XL10-Gold	BL21 Gold (DE3)
Plasmid isolation kit	—	Plasmid DNA Purification Kit, Eurx
Ampicillin final concentration [μg/mL]	100	150
Inoculation medium	LB	LB
Preculture incubation	14 to 16 h, 20 °C, 200 rpm	18 h, 37 °C, 200 rpm
Large scale medium	NZY auto-induction	LB
Culture volume [L]	0.5	6 × 1
OD_600_ value	0.6	1.0
IPTG final concentration [mM]	1	0.5
Large scale cultivation	14 to 16 h, 20 °C, 200 rpm	18 h, 20 °C, 180 rpm
Cell harvesting	4 °C, 6,000 × *g*, 30 min	4 °C, 4,500 × *g*, 15 min
Cell resuspension buffer	20 mm NaH_2_PO_4_, 300 mm NaCl, 20 mm imidazole pH 8.0	50 mm HEPES-NaOH pH 7.5, 500 mm NaCl, 20 mm imidazole, 2 mm TCEP [tris(2-carboxyethyl)phosphine]
Cell lysis	French press: 2 passes, 1,100 psi Sonication: 10 × 30 s, 15 s pause	Sonication: 5 min total (4 s pulse, 26 s pause)
Removal of unlysed material	4 °C, 36,000 × *g*, 30 min	4 °C, 20,000 × *g*, 30 min
Wash/binding buffer	50 mm NaH_2_PO_4_, 300 mm NaCl, 40 mm imidazole pH 8.0	50 mm HEPES-NaOH pH 7.5, 500 mm NaCl, 20 mm imidazole, 2 mm TCEP
Elution buffer	50 mm NaH_2_PO_4_, 300 mm NaCl, 250 mm imidazole pH 8.0	50 mm HEPES-NaOH pH 7.5, 500 mm NaCl, 400 mm imidazole, 1 mm TCEP
Method for imidazole/TEV removal	Amicon Ultra-15 Centrifugal Filter, 30 kDa molecular weight cutoff, 3 cycles of dilution/concentration in 100 mm MOPSO pH 7.0, 150 mm NaCl	Dialysis in buffer: 50 mm HEPES-NaOH pH 7.5, 500 mm NaCl, 1 mm TCEP
SEC buffer	100 mm MOPSO pH 7.0, 150 mm NaCl	50 mm HEPES-NaOH pH 7.5, 50 mm NaCl, 1 mm TCEP
Determination of protein concentration	Bradford assay	Nanodrop
Freezing and storage	Glycerol at final concentration 20%, liquid nitrogen, −70 °C	Liquid nitrogen, −70 °C
PvBSAS4;1 final concentration [mg/mL]	0.35	33.0

Briefly, *E. coli* cells were transformed by the heat shock method with the pQE-30 vector containing the PvBSAS4;1 construct. A single colony was used for the inoculation of 5 mL of LB medium supplemented with a final concentration of 100 μg/mL ampicillin. The minipreps were incubated for 18 h at 37 °C, with agitation of 180 revolutions per minute (rpm), and the plasmids were isolated using commercially available kits. To confirm the correctness of cloning, the isolated plasmids were sequenced by external companies. The correct clones were used for inoculation of LB precultures, which were then incubated for 18 h at 37 °C and 180 rpm. Next, the precultures were used for inoculation of the final cultures (10 mL/L), grown in different media. When cells reached OD_600_, protein expression was induced with isopropyl β-D-1-thiogalactopyranoside (IPTG). After overnight incubation, cells were harvested by centrifugation, resuspended in buffer, and lysed. PvBSAS4;1 was purified by His-tag affinity chromatography using Ni-NTA agarose resin, followed by size-exclusion chromatography on a HiLoad Superdex 200 16/600 column. The size and purity of the eluted protein were assessed by SDS-PAGE. Before snap-freezing in liquid nitrogen, protein concentration was measured according to Bradford assay (Bradford 1976) or at 280 nm wavelength using a NanoDrop spectrophotometer, with the following protein parameters calculated from the protein sequence (Wilkins et al. 1999): molar extinction coefficient *ε* = 20,650 m^−1^ cm^−1^ and molecular weight 34,513 Da. Protein solutions were aliquoted (100 µL) and stored at −70 °C for enzymatic or crystallographic studies.

### Crystallization

PvBSAS4;1 was crystallized using vapor diffusion in a sitting-drop setup at 19 °C. The protein crystallized in the F7 condition of the BCS screen from Molecular Dimensions (Chaikuad et al. 2015), containing 0.2 m MgCl_2_, 0.1 m Tris–HCl pH 8.0, 25% (v/v) PEG smear high, and 10% glycerol, enriched with additional glycerol up to the final concentration of 20% for cryoprotection. The protein was mixed with the F7 solution in a 3:1 ratio (1.5 µL:0.5 µL); the reservoir contained 60 µL of the precipitant solution. Crystals appeared after 2 d and were harvested using cryo-loops (Molecular Dimensions), flash-frozen in liquid nitrogen, and stored for X-ray data collection.

### Determination and refinement of the crystal structure

Diffraction data were measured at the P13 beamline of the PETRA III synchrotron in Hamburg, Germany. The crystal structure was solved by molecular replacement (MR) in *Phaser* (Mccoy et al. 2007), with chain A of the crystallographic model of *O*-acetylserine sulfhydrylase (PDB ID: 1Z7W) from *Arabidopsis thaliana* as the search model, prepared for MR in *Phenix.sculptor* (Bunkoczi and Read 2011). The preliminary model was standardized in the asymmetric unit using *ACHESYM* (Kowiel et al. 2014). The model of PvBSAS4;1 was refined in *Phenix.refine* (Afonine et al. 2012) and rebuilt in electron density maps in *Coot* (Emsley et al. 2010). The omit map was generated using *Phenix.composite_omit_map* (Terwilliger et al. 2008). The structure was refined isotropically with translation-libration-screw (TLS) ADP parameters. Ligand restraints for PLP and BEZ were generated in *eLBOW* (Moriarty et al. 2009).

### Molecular docking and MD simulations

In silico docking studies were performed in *Glide* (Friesner et al. 2004) within *Maestro* 2025-1 (Schrödinger, LLC, New York, NY, 2025). The protein chain was prepared for docking, starting from the crystal structure of this work; explicit water molecules from the crystal structure were retained. The N- and C-termini were built to include the entire sequence of PvBSAS4;1 for optimal representation during subsequent MD simulations. The ligands (free OAS and PLP–OAS aldimine) were prepared using LigPrep (Schrödinger, LLC, New York, NY, 2025). The docking grids were centered at the position of the BEZ ligand. To obtain a complex with free OAS, no further modifications were performed. To model a complex with free Lys and PLP–OAS as an external aldimine, PLP was removed from the crystal structure prior to docking. In both cases, only the highest-scoring docking poses were retained.

Prior to MD simulations, the individual subunits resulting from docking were copied by the crystallographic 2-fold axis in *Coot* (Emsley et al. 2010) to reconstruct the physiological dimers. The MD simulations were performed in *Desmond 8.1.129* (Bowers et al. 2006) using *Maestro* 2025-1 (Schrödinger, LLC, New York, NY, 2025). Each system was prepared using the latest OPLS5 force field (Lu et al. 2021) and the TIP4P water model. Explicit water molecules from the crystal structure were retained. To maintain charge neutrality, Na⁺/Cl⁻ ions were added, along with additional salt to achieve a 150 mm NaCl concentration. Standard relaxation procedure was carried out before the production runs. MD simulations were conducted for 1,000 ns at 300 K under NPT conditions (constant number, pressure, and temperature). Trajectory snapshots were saved every 1 ns, yielding 1,000 frames per system. Two replicas per system were run using randomized velocities. Ligand trajectories were analyzed independently in the replicas and subunits using the *Simulation Interactions Diagram* tool in *Maestro*.

### Other software

Surface potential was calculated using the *PDB2PQR* server (Dolinsky et al. 2004). The electrostatic potential of the surface is expressed in the units of *kT*/*e* = *k*_B_*T*/*e*_c_ = 25.6 mV at 298 K, where *k*_B_ is the Boltzmann constant, *T* is absolute temperature in kelvins, and *e*_c_ is the elementary charge (Jurrus et al. 2018). Models were visualized in *ChimeraX* 1.8 (Pettersen et al. 2021; Meng et al. 2023) and *Chimera* 1.15 (Pettersen et al. 2004). Polder maps were generated in *Phenix.polder* (Liebschner et al. 2017). Protein sequences were obtained from UniProt (Bateman et al. 2021). The topology diagram was generated using PDBsum (Laskowski et al. 2017). Multiple sequence alignment was calculated in MEGA (Tamura et al. 2021).

### Enzymatic assay

To determine the inhibitory effect of benzoic acid on PvBSAS4;1 enzyme activity, an assay was performed in the presence or absence of benzoic acid. A broad concentration range (10 μM to 2 mm) of benzoic acid was used at the beginning to determine the optimal concentration for inhibition. A reaction was performed in 100 µL of 100 mm 2-hydroxy-3-morpholinopropanesulfonic acid (MOPSO) buffer (pH 7.0), with 8 mm OAS and 1.5 mm sodium sulfide, incubated for 20 min at 25 °C. Two hundred nanograms of enzyme were added. The negative control was a reaction without enzyme; the positive control was without benzoic acid. Acid ninhydrin reagent was prepared by dissolving 250 mg of ninhydrin in 6 mL of glacial acetic acid and 4 mL of concentrated HCl. The reaction was stopped by adding 50 µL of 40 mm HCl premixed with an equimolar amount of acid-ninhydrin reagent. As described by Gaitonde (1967), the acid-ninhydrin reagent reacts specifically with cysteine, forming a colored product. The production of cysteine was measured with a PowerWave XS plate reader (Fisher Scientific, Ottawa, Ontario, Canada) at 560 nm. Enzyme kinetic assays were performed to determine the half-maximal inhibitory concentration (IC_50_) and the type of inhibition. Reactions were prepared with a series of substrate concentrations (2 to 8 mm of OAS; 1.5 mm of sodium sulfide) in the presence of inhibitors at 0 to 2 mm concentration. For enzyme kinetics, a total of 16 assays were performed at once for accuracy. A Lineweaver–Burk plot was used to determine the type of inhibition by benzoic acid by looking at the *V*_max_ of the reactions with the change of substrate concentration. Other compounds with molecular structure similar to benzoic acid, including salicylic acid, tyrosine, and phenylalanine, were also used in this assay as inhibitor candidates. The IC_50_ values were calculated as follows. A linear regression was used for data points between concentrations of 0 and 1.2 mm benzoic acid or salicylic acid, which represent the linear portion of the curve, after which the activity reached a minimum. The midpoint of the range of activity was determined as the arithmetic mean of the 2 values of *v* at 0 and 1.2 mm concentration. The following regression equation was used to calculate the IC_50_:


$$y\;=\;ax\;+\;b,\;\mathrm{where}\;IC_{50}=(\mathrm{midpoint}\text{–}b)/a$$

### Plant material and growth conditions

The genotype BAT93 of common bean (*P. vulgaris*) was grown in a growth cabinet (Conviron, Winnipeg, Manitoba, Canada) with a cycle of 16 h light (300 to 400 μmol photons m^−2^ s^−1^) at 24 °C and 8 h dark at 18 °C. Seeds were germinated in 8 cm × 10 cm pots with vermiculite, and seedlings were transplanted to larger pots (17 cm × 20 cm) with Promix BX soil (Premier Tech Horticulture, Rivière-du-Loup, Québec, Canada). Plants were fertilized with 0.75 g of 20:20:20 (N:P:K) fertilizer per pot once a week after transplantation.

### Embryo culture and culture media

Pods were harvested 12 to 15 d after fertilization. Pods were surface-sterilized with 0.5% bleach and a few drops of diluted soap (Alconox powdered precision cleaner, NY, United States), followed by 3 washings in sterile Milli-Q water for 5 min. Developing seeds between 20 and 36 mg were selected, their seed coats removed, and the cotyledons transferred to 6-well surface-treated culture plates (VWR International, Mississauga, Ontario, Canada). This was performed in a laminar flow hood (Bio-Klone 2, Microzone Corporation). Each well contained 2.5 mL of filter-sterilized growth media and 6 cotyledons. Culture plates were incubated under continuous light with shaking at 60 rpm for 48 h at room temperature. Basic culture media composition followed Joshi et al. (2019) with a minor change, and contained 8 mm MgSO_4_, 10 mm KCl, 3 mm CaCl_2_, 1.25 mm KH_2_PO_4_, 0.5 mm MnSO_4_, 0.15 mm ZnSO_4_, 0.1 mm ethylenediaminetetraacetic acid (EDTA) iron (III) sodium salt, 0.1 mm H_3_BO_3_, 27 μM glycine, 2.5 μM CuSO_4_, 5 μM KI, 1 μM Na_2_MoO_4_, 0.1 mm CoCl_2_, 4 μM nicotinic acid, 1 μM thiamine-HCl, 0.5 μM pyridoxine-HCl, 0.56 mm myo-inositol, 0.1 mm Na_2_EDTA, 5 mm 2-(*N*-morpholino)ethanesulfonic acid (MES), 146 mm sucrose, and 62.5 mm glutamine. The pH of the culture media was adjusted to 5.8 with KOH. To determine the optimal concentration of benzoic acid for in vitro incubation, a series of benzoic acid concentrations (0 to 1.2 mm) was tested, and the appearance and growth in fresh weight of cotyledons were recorded after incubation for 48 h. The basic media were supplemented with 8 mm  ^13^C_3_,^15^N-serine and 1.2 mm benzoic acid. For experiments designed to track the incorporation of ^13^C-labeled methyl group from sodium thiomethoxide, there were 3 treatment groups: labeled sodium thiomethoxide supplementation, nonlabeled sodium thiomethoxide supplementation, and no supplementation. Compounds were supplied at a concentration of 8 mm. Cotyledons were washed 3 times with filter-sterilized Milli-Q water to remove excessive labeled compounds from the surface. Seeds were dried with Kimwipes and stored at −80 °C after flash freezing in liquid nitrogen for future analysis.

### Amino acid extraction and MS analysis

To measure the level of isotope-labeled compounds after incubation, amino acids were extracted from frozen cotyledons in extraction buffer (50 mm ammonium bicarbonate in ethanol, pH 5.6) and processed for mass spectrometry (MS) analysis. The frozen cotyledons were homogenized using TissueLyser II (Qiagen Inc.) with ceramic beads in 1.5 mL Eppendorf tubes. Extraction buffer was added to ground seeds, followed by vortexing and incubation in a sonic water bath at 40 kHz to extract soluble metabolites. Samples were treated with 2 mm dithiothreitol (DTT) for 30 min at 45 °C to break disulfide bonds formed between metabolites. Because disulfide bonds are reversible, 6 mm of N-ethylmaleimide (NEM) (Heinemann and Hildebrandt 2021) was added to samples to prevent S–S bond re-formation. To quench the remaining NEM, an additional 2 mm DTT was added. The supernatant was collected after centrifugation at 10,000 × *g* and 4 °C for 10 min and transferred to a 2 mL amber glass vial (Agilent Technologies) after filtration through a 0.45 μm polytetrafluoroethylene syringe filter. MS data were obtained with a Q-Exactive Orbitrap mass spectrometer (Thermo Fisher Scientific, https://www.thermofisher.com/ca/en/home.html) coupled to an Agilent 1290 HPLC system. Methods for MS/MS and LC-data-dependent acquisition experiments, along with the method for data analysis with the XcaliburTM software, were followed as described (Joshi et al. 2019). The incorporation percentage of labeled compounds was calculated by determining the ratio of signal intensity of labeled compounds to the sum of labeled and unlabeled signal intensity.

### Chemicals and media

L-Serine (^13^C_3_, 99%; ^15^N, 99%) was manufactured by Cambridge Isotope Laboratories, Inc. (Andover, MA, United States). ^13^C-Labeled sodium thiomethoxide (sodium methanethiolate-13C) was purchased from Toronto Research Chemicals (Toronto, Ontario, Canada). Other chemicals were from Sigma-Aldrich (Mississauga, Ontario, Canada).

## Supplementary Material

kiaf485_Supplementary_Data

## Data Availability

Atomic coordinates and structure factors are available from the Protein Data Bank (Burley et al. 2019) under the accession code 9RJ1. Raw X-ray diffraction data have been deposited in the Macromolecular Xtallography Raw Data Repository (https://mxrdr.icm.edu.pl/) under the following DOI: https://doi.org/10.60884/BCOLCQ.

## Supplementary data

The following materials are available in the online version of this article.

**Supplementary Figure S1.** Topology diagram of PvBSAS4;1. Red cylinders represent α-helices, and pink arrows represent β-strands.

**Supplementary Figure S2.** Multiple sequence alignment of BSAS4;1 with its plant, bacterial, and protozoan homologs.

**Supplementary Figure S3.** Analysis of the MD simulations.

**Supplementary Figure S4.** Schematic representation of PvBSAS4;1 interactions with PLP–OAS external aldimine during the MD simulation.

**Supplementary Figure S5.** Details of the inhibition brought about by benzoic acid.

**Supplementary Figure S6.** Inconsistent locations of OAS mapped on the 3D structures of homologs of PvBSAS4;1.

**Supplementary Figure S7.** Inconsistent interpretations of OAS positioning in PDB homologs of PvBSAS4;1.

**Supplementary Table S1.** Summary of sequence and structure identities between PvBSAS4;1 and selected homologs.

**Supplementary Table S2.** Summary of structural identities between PvBSAS4;1 and homologous structures containing the OAS ligand.
