# Supplementary material for: Benzoic and salicylic acids inhibit β-substituted alanine synthase 4;1 in common bean
Source: Plant Physiol. 2025 Oct 6;199(3):kiaf485. doi: 10.1093/plphys/kiaf485 (PMC12626222; doi:10.1093/plphys/kiaf485)
Supplement: kiaf485_Supplementary_Data [file kiaf485_supplementary_data.pdf]

Supplementary Figures and Tables

Benzoic and salicylic acids inhibit β-substituted alanine synthase 4;1 in common bean

Zixuan Lu[1,2*], Wojciech Witek[3*§✉], Milosz Ruszkowski[3✉], Barbara Imiolczyk[3], Nataliya Paulish[3], Jaya Joshi[1,2,4], Mariusz Jaskolski[3,5], Frédéric Marsolais[1,2✉]

[1] Agriculture and Agri-Food Canada, London Research and Development Centre, London, Ontario, Canada;
[2] Department of Biology, University of Western Ontario, London, Ontario, Canada;
[3] Department of Structural Biology of Eukaryotes, Institute of Bioorganic Chemistry, Polish Academy of Sciences, Poznan, Poland;
[4] Department of Wood Science, University of British Columbia, Vancouver, British Columbia, Canada
[5] Department of Crystallography, Faculty of Chemistry, Adam Mickiewicz University, Poznan, Poland
[*] Equal contribution
[§] Current affiliation: Membrane Structural and Functional Biology Group, School of Biochemistry and Immunology, Trinity College Dublin, Dublin, Ireland
✉ Corresponding authors: Wojciech Witek, E-mail: witekw@tcd.ie ; Frédéric Marsolais, Frederic.Marsolais@agr.gc.ca ; Milosz Ruszkowski, mruszkowski@ibch.poznan.pl
The author responsible for distribution of materials integral to the findings presented in this article in accordance with the policy described in the Instructions for Authors (https://academic.oup.com/plphys/pages/General-Instructions) is Frédéric Marsolais (Frederic.Marsolais@agr.gc.ca).

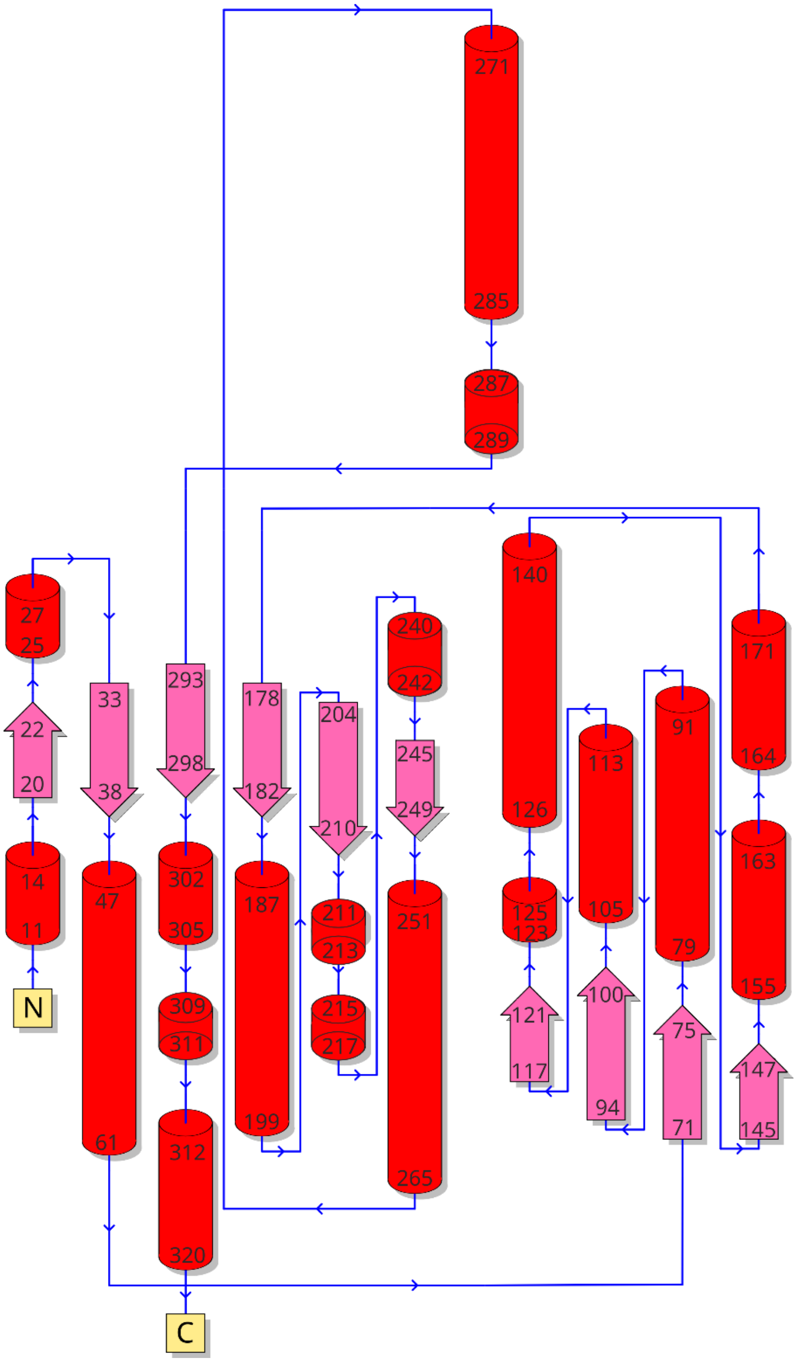

Supplementary Figure S1. Topology diagram of *Pv*BSAS4;1. Red cylinders represent α-helices, pink arrows represent β-strands.

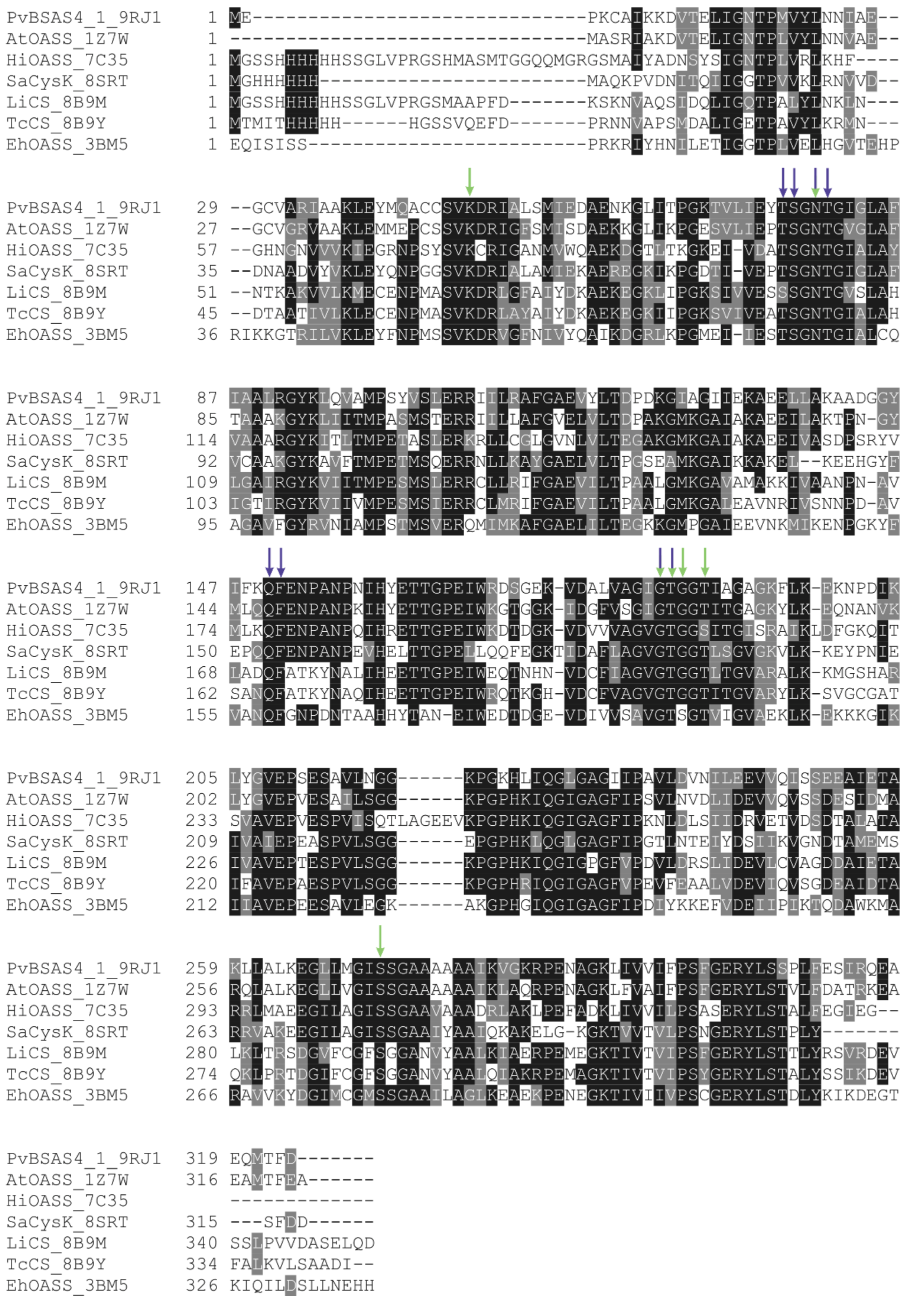

Supplementary Figure S2. Multiple sequence alignment of BSAS4;1 with its plant, bacterial, and protozoan homologs. The green arrows indicate the active site residues interacting with PLP and purple arrows indicate those interacting with benzoate, while double-colored arrows indicate residues interacting with both ligands. Gray and black boxes show similar and identical residues, respectively. The sequences are encoded in the following order: species, enzyme, PDB code. Pv = *Phaseolus vulgaris*, At = *Arabidopsis thaliana*, Hi = *Haemophilus influenzae*, Sa = *Staphylococcus aureus*, Li = *Leishmania infantum*, Tc = *Trypanosoma cruzi*, Eh = *Entamoeba histolytica*.

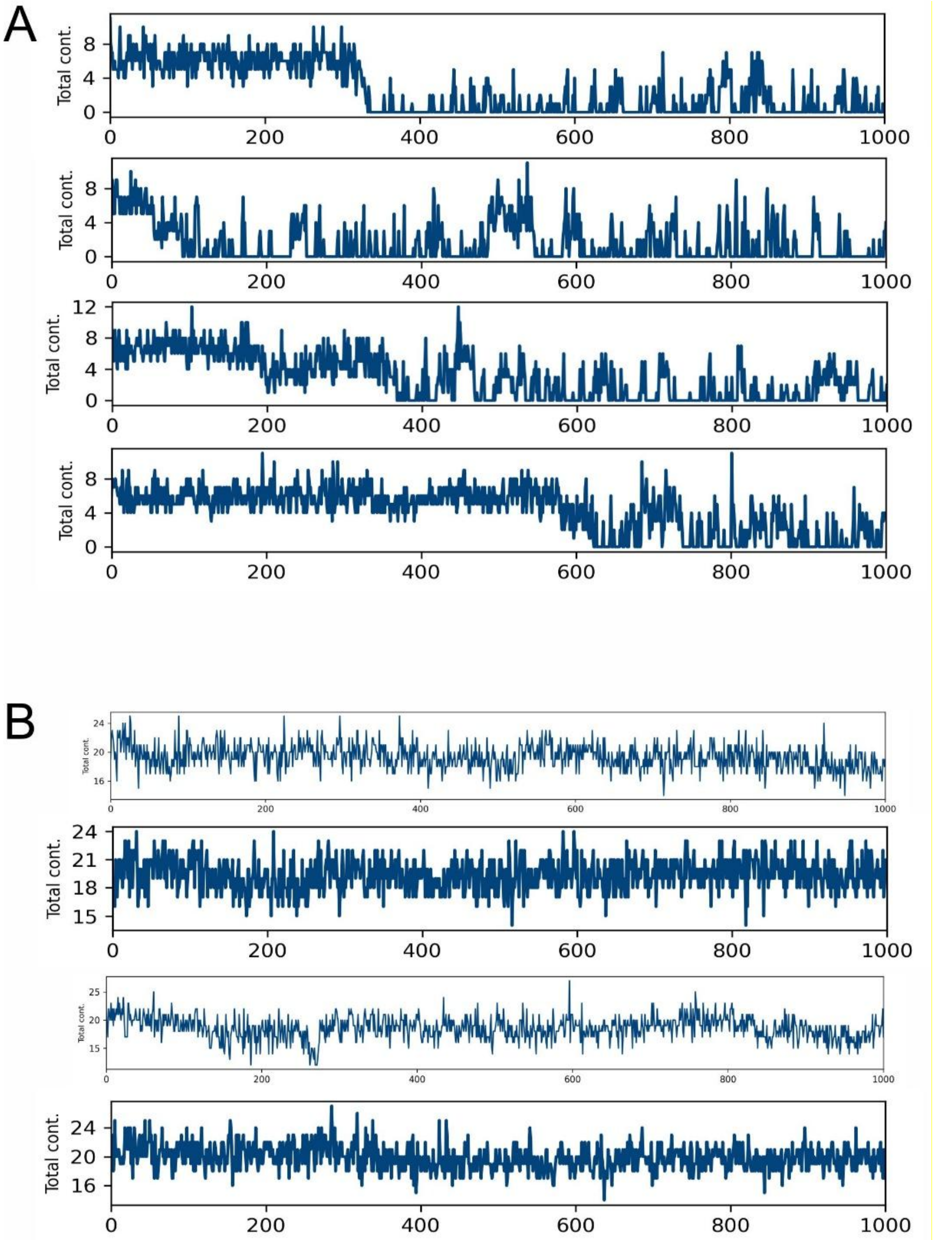

Supplementary Figure S3. Analysis of the Molecular dynamics simulations. (A and B) timeline representations of contacts (H-bonds, Hydrophobic, Ionic, Water bridges) the protein makes with the ligand over the course of the trajectory. Panel (A) represents the *in silico* model of PvBSAS4;1 as the internal PLP aldimine complexed with free OAS. The number of contacts formed between the PLP-OAS external aldimine and PvBSAS4;1 with free Lys48 is plotted in panel (B). Each system contained the obligate dimer, and the simulation was performed in duplicate, yielding four events per system.

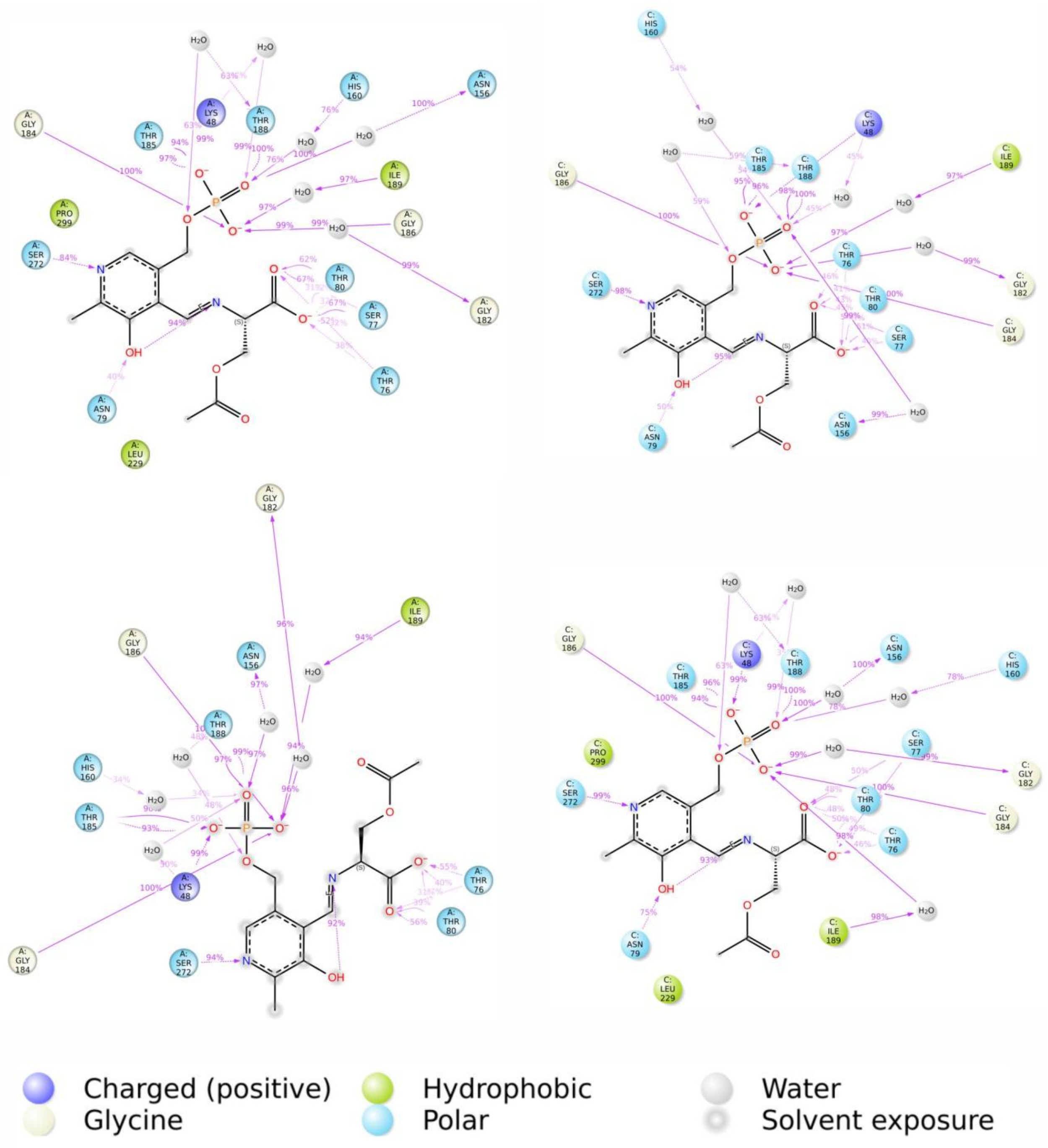

Supplementary Figure S4. Schematic representation of PvBSAS4;1 interactions with PLP-OAS external aldimine during the MD simulation. Only interactions persisting for more than 30% of the simulation time in the selected trajectory (0–1000 ns) are shown. Types of interactions are color-coded according to the legend at the bottom. The order of illustrations corresponds to that in Supplementary Figure S3B.

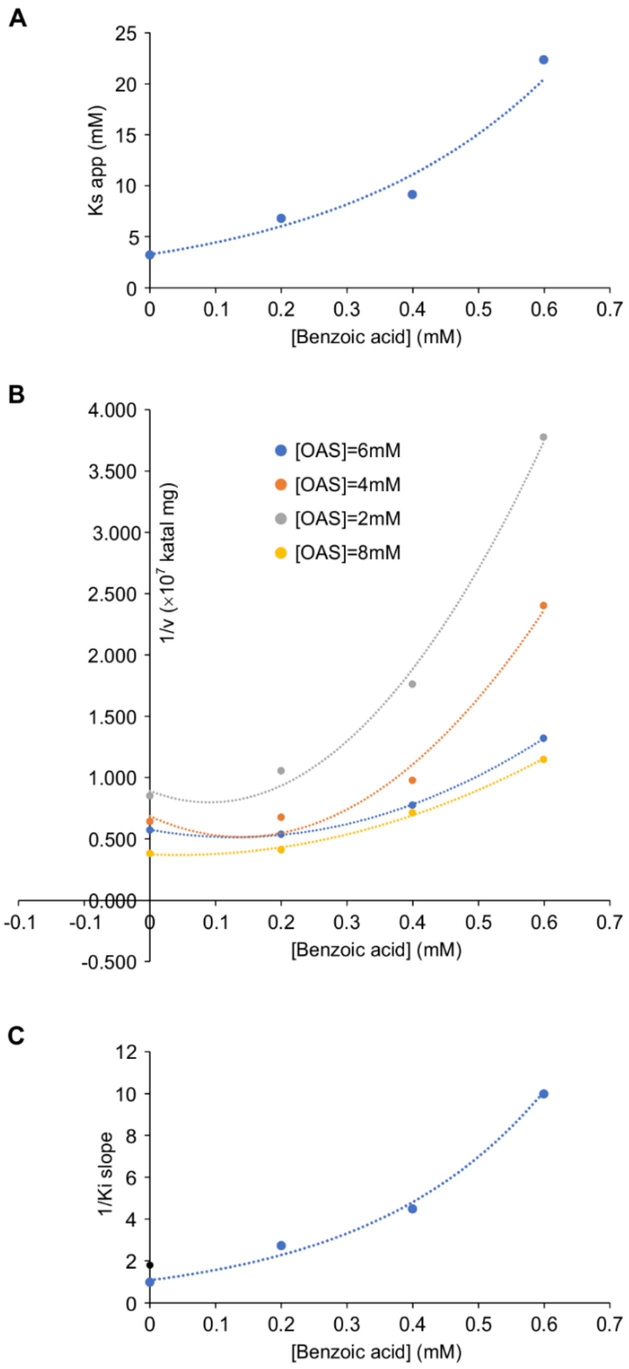

Supplementary Figure S5. Details of the inhibition brought about by benzoic acid. (A) Replot of $K_{s\ app}$ versus the concentration of benzoic acid. (B) Dixon plot of the reciprocal of the rate versus the concentration of benzoic acid. (C) Replot of the reciprocal of $K_{i\ slope}$ versus the concentration of benzoic acid.

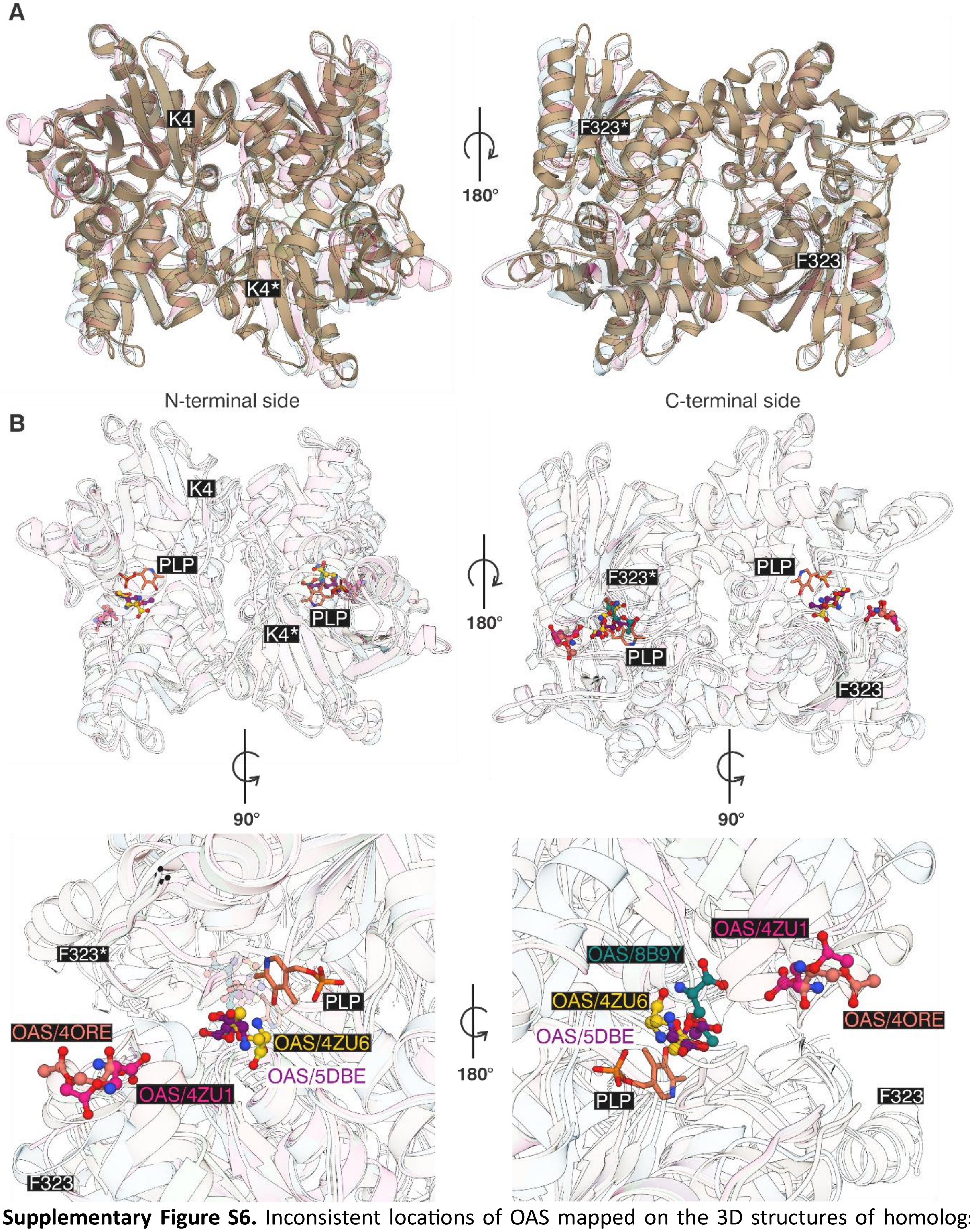

Supplementary Figure S6. Inconsistent locations of OAS mapped on the 3D structures of homologs of PvBSAS4;1. Panel (A) depicts a superposition of PvBSAS4;1 with 4ORE, 4ZU1, 4ZU6, 5DBE, and 8B9Y. PvBSAS4;1 is in coral, whereas the homologous structures are 95% transparent. Panel (B) depicts the variability of OAS modelling in the PDB deposited structures. PLP marks the location of the active site. The OAS molecules are colored and labeled according to the PDB codes: salmon 4ORE, gold 4ZU6, pink 4ZU1, magenta 5DBE, dark cyan 8B9Y.

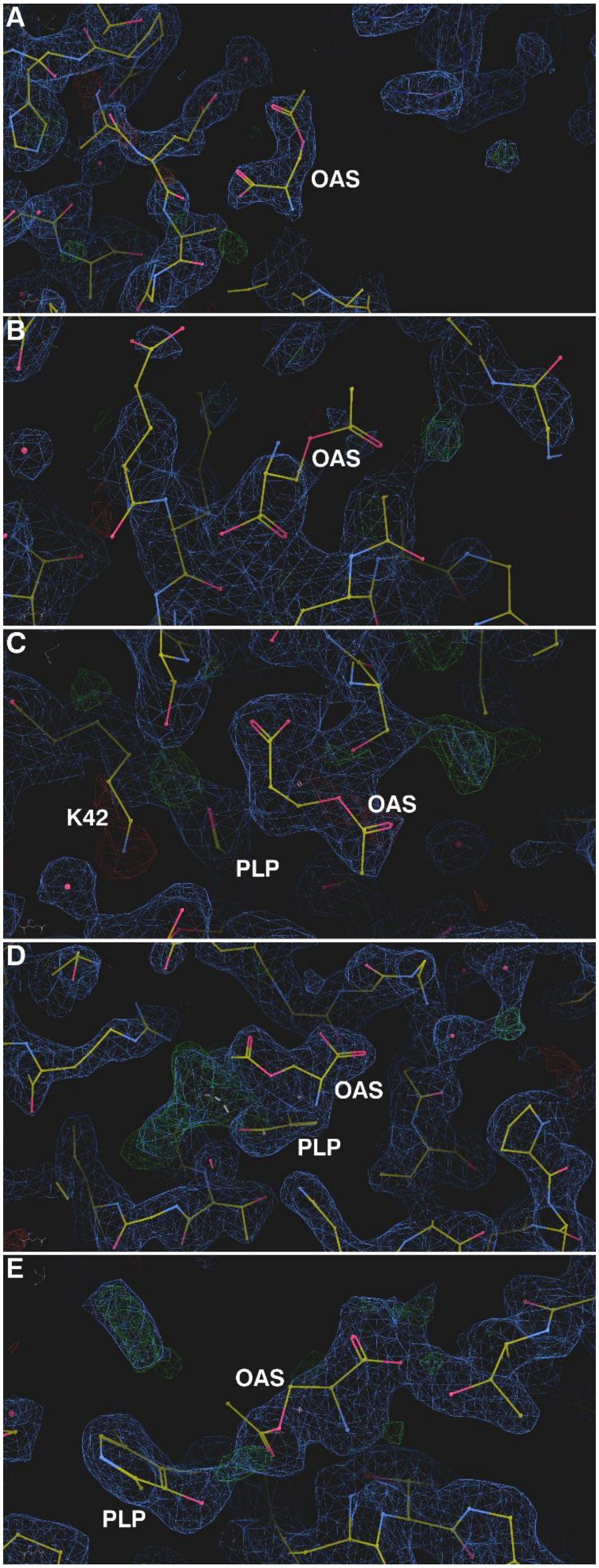

Supplementary Figure S7. Inconsistent interpretations of OAS positioning in PDB homologs of PvBSAS4;1. All 2Fo-Fc electron density maps (blue) are contoured at 1.0σ, and difference Fo-Fc maps at 3.0σ (green positive, red negative). (A) OAS in entry 4ORE. The entry has no information about the presence of OAS in the crystallization condition. (B) OAS modeled on the protein surface in 4ZU1 despite the absence of convincing electron density. (C) Electron density in 4ZU6 indicates that PLP should create an internal aldimine with lysine 42. (D) Unmodeled key residues involved in OAS binding in 5DBE, despite a very strong difference in electron density. (E) Inverted orientation of OAS in the active site of 8B9Y. The molecule should be rotated by 180° so that the amine would face PLP prior to the reaction.

Supplementary Table 1. Summary of sequence and structure identities between PvBSAS4;1 and selected homologs.

Homologous protein (PDB)	Sequence identity to PvBSAS4;1 [%]	Root mean square deviation [Å; # Cα atoms]
Arabidopsis thaliana OASS (1Z7W)	69	0.79; 289
Haemophilus influenzae OASS (7C35)	45	0.79; 261
Staphylococcus aureus CysK (8SRT)	46	0.80; 254
Leishmania infantum CS (8B9M)	41	0.93; 234
Trypanosoma cruzi CS (8B9Y)	44	0.89; 258
Entamoeba hystolytica OASS (3BM5)	39	0.80; 259

Supplementary Table 2. Summary of structural identities between PvBSAS4;1 and homologous structures containing the OAS ligand.

Homologous protein (PDB)	Root mean square deviation [Å; # Cα atoms]	Distance between OAS Cα to BSAS4;1 PLP C4' [Å]
Haemophilus influenzae OASS (4ORE)	0.79; 256	15.39
Haemophilus influenzae OASS (4ZU1)	0.76; 260	13.97
Haemophilus influenzae OASS (4ZU6)	0.77; 263	2.20
Haemophilus influenzae OASS (5DBE)	0.80; 260	3.31
Trypanosoma cruzi CS (8B9Y)	0.89; 258	5.51
